# *Bacillus cereus* Bloodstream Infection in a Preterm Neonate Complicated by Late Meningitis

**DOI:** 10.1155/2012/358789

**Published:** 2012-08-09

**Authors:** Toshinobu Horii, Kiyoko Tamai, Shigeyuki Notake, Hideji Yanagisawa

**Affiliations:** ^1^Department of Infectious Diseases, Hamamatsu University School of Medicine, 1-20-1 Handayama, Higashi, Hamamatsu, Shizuoka 431-3192, Japan; ^2^Miroku Medical Laboratory Inc., 659-2 Innai, Saku, Nagano 384-2201, Japan

## Abstract

Central nervous system infections caused by *Bacillus cereus* have rarely been reported in infants. In this paper, the case of a 2-month-old low-birth-weight female who developed meningitis 45 days after resolution of a bloodstream infection (BSI) is described. The pulsed-field gel electrophoresis results revealed that the patterns of both *B. cereus* isolates responsible for the acute meningitis and for the prior bacteraemic episode were closely related. Although the source of the infection from within the patient was not clear, it is suggested that the *B. cereus* BSI developed in the neonate was complicated by acute meningitis.

## 1. Introduction

Central nervous system (CNS) infections caused by *Bacillus cereus *are primarily of haematogenous origin and can complicate neuroinvasive procedures [1]. *B. cereus* rarely causes CNS infections in infants, but the mortality rate in such cases is high [2, 3]. Endospores of *B. cereus *are found in various environments, including healthcare settings. The occurrence of nosocomial *B. cereus* BSIs in hospitals and in neonatal intensive care units (NICUs) has been reported [4–6]. In the present paper, a case of acute meningitis caused by *B. cereus* that occurred long after *B. cereus* bloodstream infection (BSI) in an infant is described, and it is discussed whether the episode of meningitis related to the prior BSI or was new infection.

## 2. Case Presentation

A 2-month-old low-birth-weight female (body weight, 0.8 kg) developed meningitis 45 days after resolution of a BSI. The BSI was not catheter related. The patient's blood culture results were positive for* B. cereus* (006 and 007 on days 1 and 5, resp., from the onset of the BSI). *B. cereus* group was identified phenotypically as facultatively anaerobic, endospore-forming, gram-positive rods that yielded positive results for the egg-yolk reaction and utilized d-trehalose, using a 2% egg-yolk NGKG agar plate (NGKG agar base, Nissui Pharmaceutical, Tokyo, Japan) and a BBL crystal gram-positive identification system (Nippon Becton Dickinson, Tokyo, Japan). In the isolates 006 and 007, the minimum inhibitory concentrations (MICs) determined by the Etest method are shown in Table 1. Both isolates showed good susceptibility to imipenem, meropenem, gentamicin, clindamycin, vancomycin, linezolid, and levofloxacin. After treatment with adequate doses of vancomycin and meropenem—either singly or in combination—for a total of 10 and 16 days, respectively, the signs and symptoms of BSI completely resolved and the blood cultures were sterile within 20 days. However, 45 days after discontinuation of antimicrobial therapy for the BSI event, new signs and symptoms of infection were observed, including reduced oxygen saturation and bradycardia. Laboratory test data revealed a white blood cell count of 2.4 × 10^9^/L (granulocytes, 35%, lymphocytes, 58%, and monocytes, 6%). Analysis of cerebrospinal fluid (CSF) obtained by lumbar puncture revealed a glucose level of 330 mg/L and a protein level of 3.05 g/L. *B. cereus* 008 was isolated from a CSF specimen, and the patient was diagnosed as having acute meningitis caused by *B. cereus*. No evidence of BSI was obtained because the blood cultures were sterile. In addition, the urine, stool, throat, and nose cultures failed to yield *B. cereus*. The MICs of *B. cereus* 008 are shown in Table 1. The isolate showed good susceptibility to imipenem, meropenem, gentamicin, clindamycin, vancomycin, linezolid, and levofloxacin. After treatment with adequate doses of linezolid, meropenem, and clindamycin—either singly or in combination—for a total of 31, 38, and 12 days, respectively, the meningitis was cured and the CSF cultures were sterile.

## 3. Discussion

**Figure 1:**
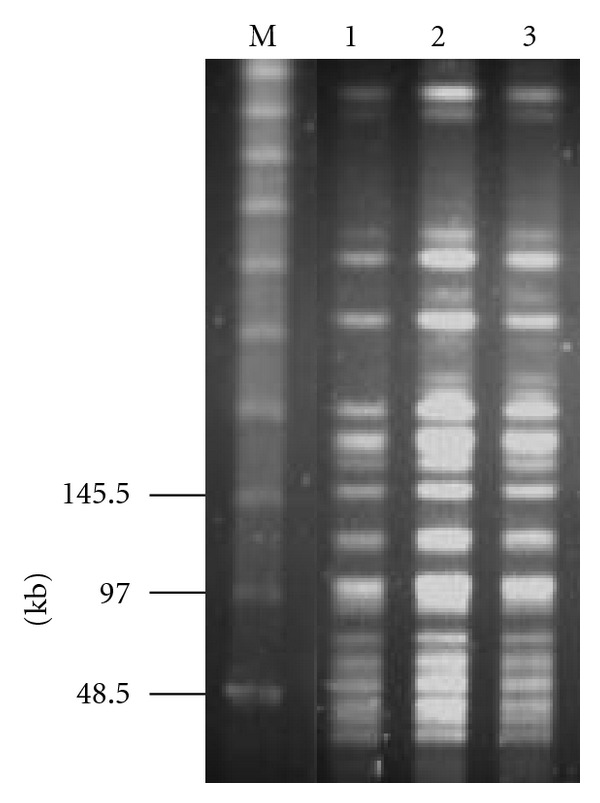
PFGE patterns of *Sma*I-restricted genomic DNA of *Bacillus cereus* isolates yielded from blood or cerebrospinal fluid culture specimens. Lane M: molecular weight marker; lane 1: *B. cereus* 006; lane 2: *B. cereus* 007; lane 3: *B. cereus* 008. Numbers on the left are DNA size markers (kb).

Transmission of *B. cereus* infections in the healthcare setting is a serious issue. *B. cereus* has been found in environmental reservoirs such as ventilator equipment, intravascular catheters, and linen. Various PFGE genotypes of *B. cereus* have been isolated from hospitals in previous studies [7]. We recently isolated several different PFGE genotypes of *B. cereus* strains from 1 piece of hospital towel from an NICU; the towel had been reused after cleaning (unpublished data). In the present study, to determine whether the episode of meningitis related to the prior BSI or was new infection, the isolates were genotyped using PFGE, as described previously [8]. In brief, an agarose gel block containing bacteria was digested with 25 U of *Sma*I for 20 h at 25°C and subjected to electrophoresis on a 1.0% agarose gel, employing a contour-clamped homogeneous electric field system (CHEF DR III, Bio-Rad Laboratories, Tokyo, Japan) at 6.0 V/cm^2^ for 18.5 h with pulse times ranging from 1.0 to 14.0 sec. For genotyping, the PFGE patterns were interpreted as described elsewhere [9]. The PFGE analysis revealed that the patterns of the 3 isolates (006, 007, and 008) were closely related (Figure 1), suggesting that the *B. cereus* strain causing the acute meningitis was epidemiologically related with the *B. cereus* strain responsible for the prior bacteraemic episode.

 In neonates, predisposing factors for *B. cereus* meningitis include low birth weight, ventricular shunts, and underlying conditions such as respiratory distress syndrome, bowel perforation, and bronchopulmonary dysplasia [2]. In the present case, no neuroinvasive procedure was performed, and she did not have any underlying condition that could have affected the development of acute meningitis except for the factor of low birth weight. The antimicrobials used before the patient developed acute meningitis included vancomycin and meropenem.

 Thus, in this case, it is likely that the *B. cereus* BSI developed in the preterm neonate was complicated by the acute meningitis during 2 months; however, the source of the infection from within the patient was not clear. The present report has another limitation because CNS infection was not considered through lumbar puncture and CSF cultures at the time the blood cultures were first positive. In conclusion, this case emphasizes that it is critical to rapidly diagnose and completely treat *B. cereus* BSIs to prevent complicated infections such as meningitis in infants.

## Figures and Tables

**Table 1:** Antimicrobial susceptibilities in *Bacillus cereus* isolates.

Antimicrobial	Minimum inhibitory concentration (mg/L)		
	006	007	008
Ampicillin	16	32	16
Ceftazidime	>256	>256	>256
Imipenem	0.125	0.125	0.125
Meropenem	0.064	0.125	0.064
Gentamicin	2	2	1
Clindamycin	0.5	0.5	0.5
Vancomycin	2	2	2
Linezolid	1	1	1
Levofloxacin	0.125	0.125	0.125
